# Visualization of micro-agents and surroundings by real-time multicolor fluorescence microscopy

**DOI:** 10.1038/s41598-022-17297-7

**Published:** 2022-08-04

**Authors:** Mert Kaya, Fabian Stein, Prasanna Padmanaban, Zhengya Zhang, Jeroen Rouwkema, Islam S. M. Khalil, Sarthak Misra

**Affiliations:** 1 Surgical Robotics Laboratory, Department of Biomechanical Engineering, University of Twente, 7522 NB Enschede, The Netherlands; 2 Surgical Robotics Laboratory, Department of Biomedical Engineering and University Medical Centre Groningen, University of Groningen, 9713 AV Groningen, The Netherlands; 3 Vascularization Laboratory, Department of Biomechanical Engineering, University of Twente, 7522 NB Enschede, The Netherlands

**Keywords:** Biomedical engineering, Other photonics

## Abstract

Optical microscopy techniques are a popular choice for visualizing micro-agents. They generate images with relatively high spatiotemporal resolution but do not reveal encoded information for distinguishing micro-agents and surroundings. This study presents multicolor fluorescence microscopy for rendering color-coded identification of mobile micro-agents and dynamic surroundings by spectral unmixing. We report multicolor microscopy performance by visualizing the attachment of single and cluster micro-agents to cancer spheroids formed with HeLa cells as a proof-of-concept for targeted drug delivery demonstration. A microfluidic chip is developed to immobilize a single spheroid for the attachment, provide a stable environment for multicolor microscopy, and create a 3D tumor model. In order to confirm that multicolor microscopy is able to visualize micro-agents in vascularized environments, in vitro vasculature network formed with endothelial cells and ex ovo chicken chorioallantoic membrane are employed as experimental models. Full visualization of our models is achieved by sequential excitation of the fluorophores in a round-robin manner and synchronous individual image acquisition from three-different spectrum bands. We experimentally demonstrate that multicolor microscopy spectrally decomposes micro-agents, organic bodies (cancer spheroids and vasculatures), and surrounding media utilizing fluorophores with well-separated spectrum characteristics and allows image acquisition with 1280 $\times$ 1024 pixels up to 15 frames per second. Our results display that real-time multicolor microscopy provides increased understanding by color-coded visualization regarding the tracking of micro-agents, morphology of organic bodies, and clear distinction of surrounding media.

## Introduction

The field of microrobotics has opened up new avenues for various applications in medicine thanks to the advances in micro/nano-fabrication technologies^1–3^. One of the most prominent applications is targeted drug delivery, which is an innovative technique to increase the success rate of the treatment, mitigate the side effects of the medications, and reduce patient recovery time^4,5^. Micro-agents, the end-effectors of the microrobotics systems, are utilized as carriers for nano-particle drug delivery and steered towards the tissue of interest by external stimuli (e.g., magnetic fields and acoustic waves)^6^. Imaging techniques are utilized for micro-agents to reach the target tissue since sensor integration remains a challenge due to size limitations^7,8^. Acquired images can be considered only feedback source for target identification, manipulation of the micro-agents, and releasing the drugs in the desired location. Therefore, clear visualization plays a crucial role in the delivery process.

Magnetic resonance imaging (MRI), computed tomography (CT), fluoroscopy, ultrasound, and photoacoustic imaging are used to visualize micro-agents in vitro and in vivo conditions. MRI is used for simultaneous actuation and visualization of micro-agents with a high contrast-to-noise ratio^9–11^. In addition, MRI images contain anatomical details with a high contrast-to-noise ratio for precise steering of the micro-agents. However, the low image acquisition rate of MRI makes it not suitable for micro-agent applications that require real-time visualization^12^. Similar to MRI, CT provides high-resolution images of micro-agents but it has limited working space for the integration of actuation and sensing systems^13^. Fluoroscopy is an alternative imaging method for CT to obtain a larger workspace and achieve more image acquisition rate^14,15^. Both CT and fluoroscopy have harmful effects on both clinicians and patients due to ionizing radiation exposure^16^. Among the imaging modalities, ultrasound-based techniques do not have any known side effects on health and are used for real-time visualization of the micro-agents^17–21^. Ultrasound imaging provides a large working space for the placement of the actuation systems since images are acquired using a small hand-held probe^22–24^. However, ultrasound images are inherently noisy and contain artifacts, which hinder the detection of micro-agents. Photoacoustic imaging overcomes the limitation in ultrasound imaging by contrast enhancement of micro-agents. The light absorption heats up micro-agents containing metallic materials, and subsequent acoustic waves are generated by thermal expansion^25^. The generated acoustic waves render that micro-agents achieve a higher signal-to-noise ratio than ultrasound imaging and are resolved from the surroundings^26–28^.

Optical microscopy techniques are used for preliminary tests and micro-agents in lab-on-a-chip applications since the experimental environments are fabricated with transparent materials^6,7^. Bright-field and single-band fluorescence microscopy techniques are widely-used for visualizing micro-agents. Full visualization of the samples is obtained with bright-field microscopy, but height differences between the micro-agents and physical surroundings cause blurring of the acquired images^29^. Single-band fluorescence microscopy provides only visualization of micro-agents with high-resolution and does not provide information about surroundings^30,31^. In our previous study, multicolor fluorescence microscopy was introduced to overcome the limitations in optical microscopy techniques for visualizing micro-agents^29^. A wide-field multicolor microscope was developed as a tool to collect focused images from different spectrum bands and correct aberrations. The full samples were spectrally resolved, and occlusion-free visualization of micro-agents and surroundings was obtained.

In this study, we address the lack of real-time multicolor imaging of micro-agents and surroundings. The main difference between the imaging techniques used in previous studies and our approach is that a better understanding mechanism of functionality and dynamics of the micro-agents is established by color-coding of the full samples. Since color is an essential information carrier for human perception and cognition amplification, acquired multicolor images enable clear visualization of micro-agents and surroundings^32^. The potential of multicolor microscopy is demonstrated by visualizing mobile micro-agents as surrogates for drug carriers in 3D tumor and vascularized environments. The primary contributions of this study (in the field of microrobotics) are (1) demonstration of real-time multicolor fluorescence microscopy by spectral unmixing of micro-agents, organic bodies (cancer spheroids and vessels), and microfluidic channels. (2) Developing a microfluidic chip that immobilizes a single 3D cancer spheroid at a fixed location for delivery tasks with micro-agents and multicolor image acquisition from a confined environment. (3) Real-time color-coded visualization of micro-agents inside in vitro perfusable vascular network formed on a microfluidic system. Additionally, multiple imaging experiments are conducted using micro-agents with different geometries and sizes to show our contributions.

For imaging experiments, a tumor model is created by placing cancer spheroids formed using cervical HeLa cells in the developed microfluidic chip filled with culturing medium. In vitro based microfluidic vascular network and ex ovo based chicken chorioallantoic membrane are used as models to visualize micro-agents inside vascularized environments. Multicolor images are acquired through experiments where the micro-agents are magnetically moved inside the models. Real-time multicolor image acquisition is configured based on unambiguous color-coding as well as excitation of the fluorophores with minimal time intervals. First, spectral crosstalk disables the identification of micro-agents and surroundings from a single spectrum band and their visualization with a designated color. In order to overcome the crosstalk, emitted fluorescence photons are collected from three relatively well-separated spectrum bands for individual image formation. Second, fluorophores have a limited lifespan, and the intensity of emitted fluorescence photons decreases during imaging due to photobleaching. In order to prolong the lifespan of multicolor imaging by reducing the photodamage on the fluorophores, sequential excitation strategy is used.

The benefits of simultaneous^33,34^ and sequential^35,36^ excitation strategies for multicolor fluorescence microscopy are extensively studied using cellular structures. Simultaneous excitation of the fluorophores provides image acquisition from spectrum bands without delay. However, acquired images contain significant crosstalk that disables unambiguous detection of specific microstructures or compartments. Sequential excitation of the fluorophores and synchronous triggering of the imaging sensors (i.e., spectral multiplexing) overcome the crosstalk^37,38^. The drawback of the sequential strategy is that spectrally resolved images are acquired with an equal delay, which limits the multicolor formation rate^39^. The models containing mobile and stationary micro-agents are fully resolved without crosstalk by sequential excitation of the fluorophores in a round-robin manner and synchronous image acquisition to the excitation sequence. Real-time visualization of the models is obtained by relatively fast spectral multiplexing.

**Figure 1:**
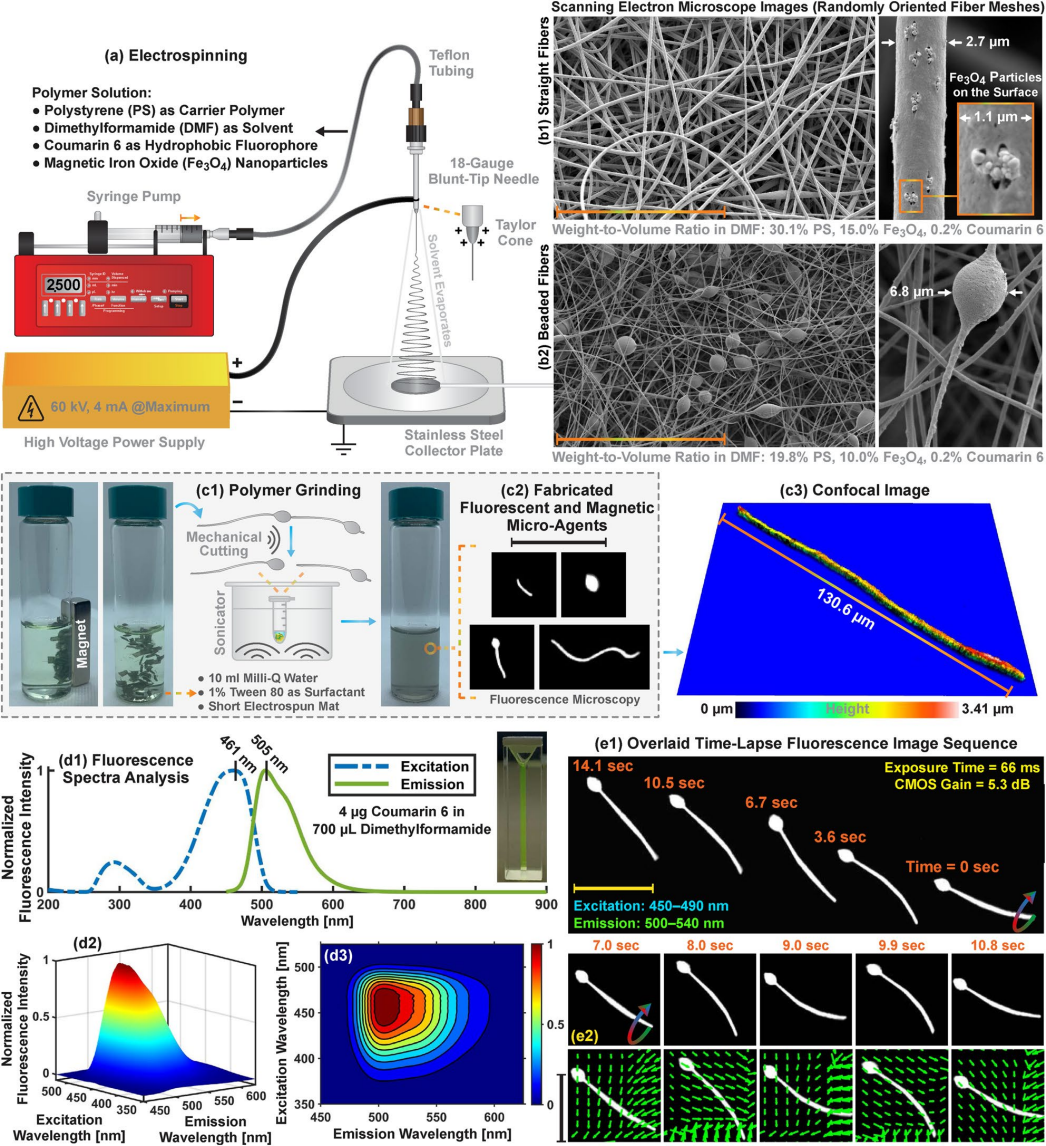
General overview for fluorescent and magnetic micro-agent fabrication using electrospinning. (**a**) Schematic representation of the electrospinning setup and content of the polymer solution. (**b1**) and (**b2**) Scanning electron microscope images of the deposited straight and beaded fiber meshes on the collector using electrospinning, respectively. (**c1**) Polymer grinding process for obtaining micro-agents by cutting the continuous fibers using a sonicator. (**c2**) Fluorescence images of the fabricated micro-agents. (**c3**) 3D surface profile of the micro-agent visualized using confocal microscopy. (**d1**) Ultraviolet-visible spectrum analysis of coumarin-6 in dimethylformamide (DMF). (**d2**) and (**d3**) Spectrum analysis of coumarin-6 in DMF at varying excitation and emission wavelengths. (**e1**) Fluorescence microscopy for visualizing micro-agent movement with a magnetic field of 21 mT. (**e2**) The vector fields overlaid on the fluorescence images show the motion of the micro-agents analyzed using Lucas–Kanade optical flow. Scale bar 100 $\upmu$m.

## Results

### Single-band fluorescence microscopy

**Figure 2:**
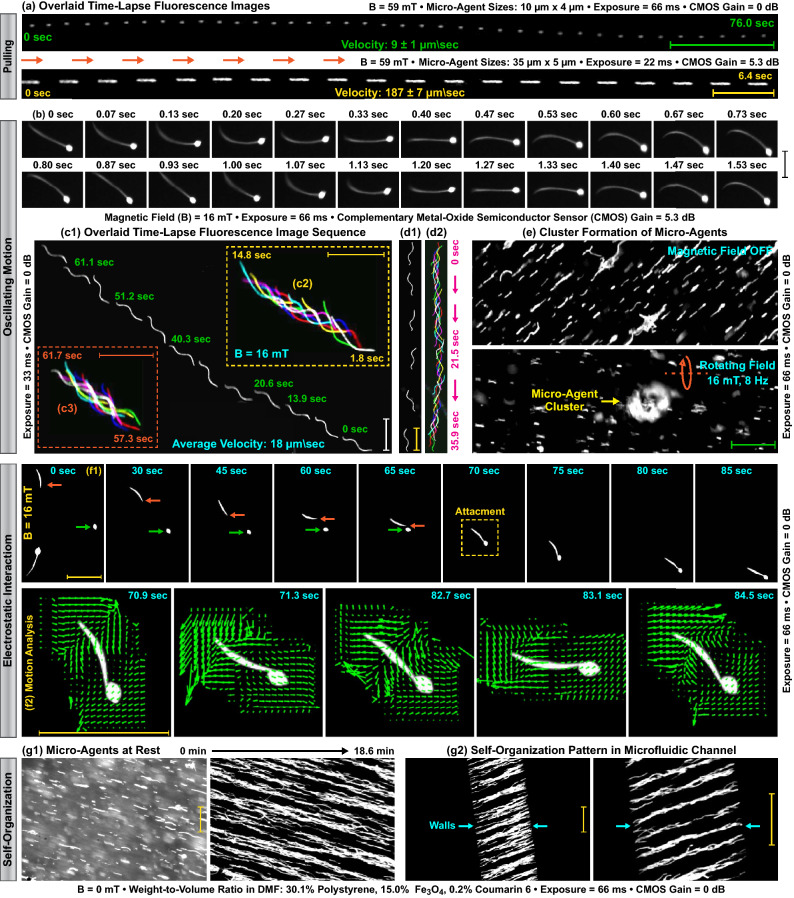
Single-band fluorescence microscopy for visualizing magnetic micro-agents labeled with coumarin 6. (**a**) Time-lapse image shows forward motion of the micro-agents by magnetic pulling. (**b**) Snapshot sequence demonstrates the oscillating motion of the micro-agent. (**c1**) and (**d1**) Overlaid fluorescence images show the diagonal and vertical movement of the micro-agents, respectively. (**c2**), (**c3**), and (**d2**) Oscillating motion patterns of the micro-agents. (**e**) Aggregation of the micro-agents by a rotating magnetic field. (**f1**) Attachment of the micro-agents by electrostatic interaction. (**f2**) Vector field expresses the motion of the attached micro-agents analyzed using Lucas-Kanade optical flow. (**g1**) and (**g2**) Self-organization of the micro-agents in a reservoir and a microfluidic channel, respectively. Scale bar 100 $\upmu$m.

Our micro-agents are fabricated by grinding the continuous beaded and straight fibers synthesized using electrospinning (Fig. 1). The synthesized fibers consist of polystyrene as a carrier polymer and iron oxide nanoparticles ($\hbox {Fe}_{3}\hbox {O}_4$) as a magnetic material (Fig. 1b). Coumarin 6 is selected to stain the fibers since it is immobilized in a polystyrene matrix and insoluble in water. Additionally, coumarin 6 has a relatively high photobleaching resistance. The grinding process breaks the fibers into fragments using ultrasound waves. This enables obtaining magnetic and fluorescent micro-agents ranging in size from 4 to 131 $\upmu$m (Fig. 1c1,c2). Single-band fluorescence microscopy is performed to visualize the motion and electrostatic interaction of the fabricated micro-agents in Milli-Q water. Figure 2a shows the pulling motion of two micro-agents fabricated using straight fibers. Figure 2b demonstrates the dynamic movement of a micro-agent fabricated using beaded fibers. During the imaging experiments, we observe that micro-agents fabricated using straight fibers are fully loaded with $\hbox {Fe}_{3}\hbox {O}_4$ and rigid. On the other hand, the polymer solution prepared for beaded fiber synthesis allows the fabrication of flexible micro-agents since the distribution of $\hbox {Fe}_{3}\hbox {O}_4$ is random. Figure 2c1–c3,d1,d2 show diagonal and vertical motion of a flexible micro-agent using an oscillating magnetic field, respectively. Electrostatic interaction between micro-agents is visualized using three experiments. The first experiment is conducted to visualize the behavior of micro-agents in a rotating field. We observe that micro-agents fabricated using both beaded and straight fibers aggregate owing to dipole-dipole interaction and form a cluster over time. Figure 2e demonstrates a representative case for a micro-agent cluster formation with a 16 mT rotating field at 8 Hz. The second experiment is devised to visualize the attachment of two micro-agents utilizing electrostatic forces. Figure 2f1 shows acquired time-lapse images by highlighting the attachment (self-assembly). The motion of the attached micro-agents is represented as vector fields using Lucas-Kanade optical flow and shown in Fig. 2f2^40^. Our results show that the attached micro-agents move together, and electrostatic forces prevent disaggregation. In the third experiment, the behavior of the micro-agents is observed when no magnetic field is applied. Acquired images reveal that micro-agents fabricated using straight fibers sink and create a line-shaped self-organization pattern in both an open reservoir and a microfluidic channel (Fig. 2g1,g2). The self-organization pattern is not observed when the experiment is repeated with the micro-agents fabricated using beaded fibers. A decrease in the magnetic material concentration in the micro-agents disables the self-organization behavior. The fabricated micro-agents are used as surrogates for drug carries and attached to HeLa cell spheroids stained with CellTracker Red CMTPX.

### Attachment of micro-agents to HeLa cell spheroids

**Figure 3:**
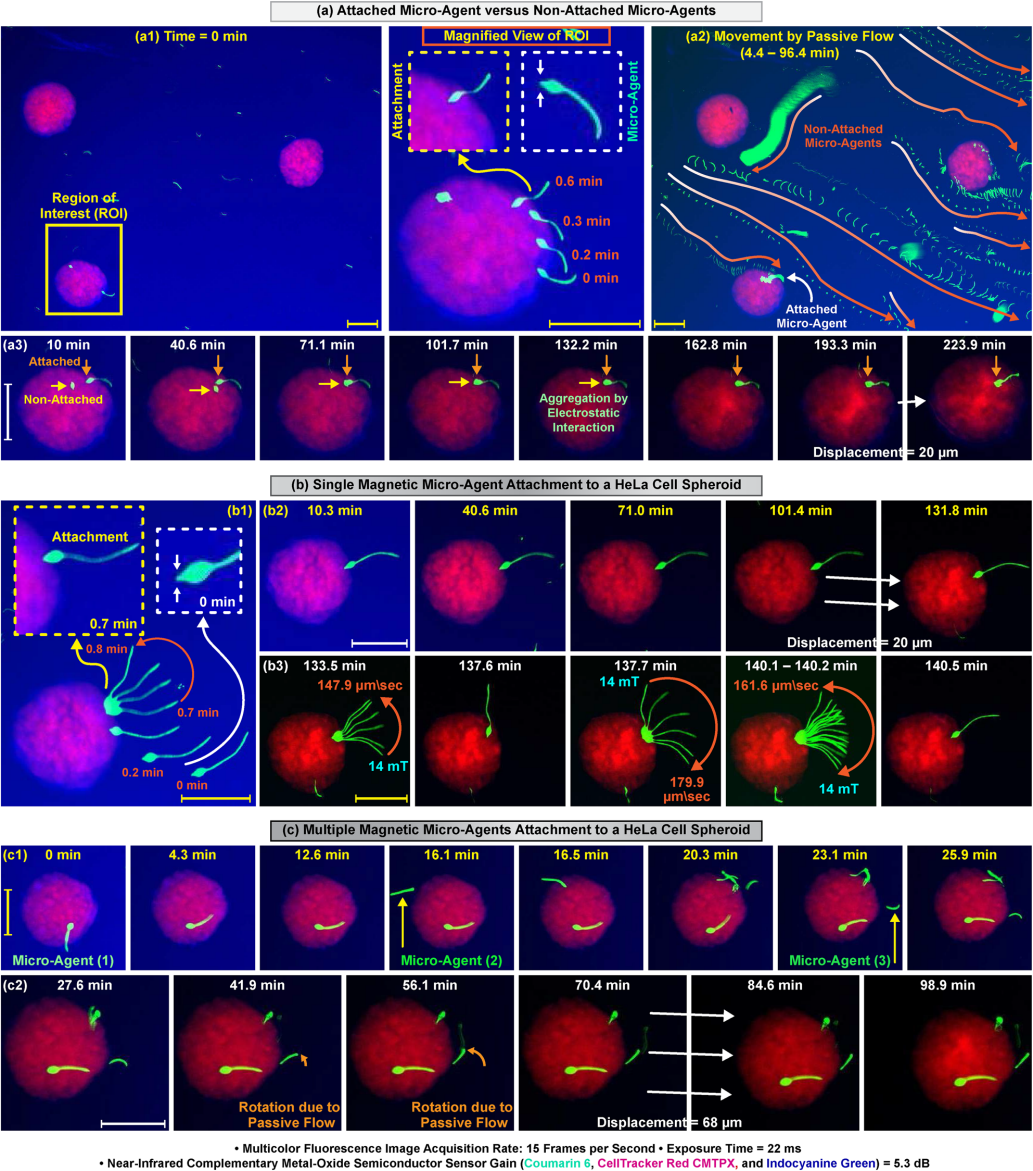
Time-lapse multicolor fluorescence microscopy for single and multiple magnetic micro-agents attachment to HeLa cell spheroids. (**a1**–**c1**) Attachment of micro-agents to HeLa cell spheroids with a magnetic field of 36 mT. (**a2**,**a3**) Effect of passive flow on the attached and non-attached micro-agents. (**b2**,**c2**) Visualization of the changes in the micro-agents attached to the spheroids. (**b3**) Attachment check by rotational movement of the micro-agent around its inserted part into the spheroid after two hours pass. Scale bar 100 $\upmu$m. Please refer to the accompanying video (Part 1).

We visualize single and multiple micro-agents attachment to HeLa cell spheroids with three experiments in an open reservoir containing indocyanine green in the culturing medium. In all experiments, multicolor images are formed at 15 fps, and micro-agents are steered towards the spheroids with a magnetic field intensity of 36 mT. In the first two experiments, micro-agents with sharp tips are attached by puncturing the spheroids (Fig. 3a1,b1). In the third experiment, three micro-agents are consecutively attached to the spheroid. The first two micro-agents are adhered to the spheroid surface by electrostatic interaction, whereas the third micro-agent is attached by puncturing (Fig. 3c1). The attachments are verified by screening long-term changes using dynamic imaging. Excitation light generates a heat gradient between the illuminated and non-illuminated areas, which results in a flow. We visualize the mobility of the micro-agents in the flow as a passive method for attachment verification. The effect of the flow on both attached and non-attached micro-agents for 92 min is displayed in Fig. 3a2. Although the velocity of the attached micro-agent is measured approximately 0 $\upmu$m/s, non-attached micro-agents are mobilized by the flow. We also observe that a non-attached micro-agent moved by the flow aggregates with the attached micro-agent utilizing electrostatic forces (Fig. 3a3). The displacement of the aggregated micro-agents is measured 0 $\upmu$m for 92 min. Our experiment validates that attachment to the spheroid increases the inertia of the micro-agents and immobilizes them in the flow. In all experiments, the attachments are verified by visualizing the micro-agents immobility in the flow for more than 90 min (Fig. 3a2–c2). Acquired dynamic images for the verification also reveal that the attached micro-agents displace along with the spheroids owing to the evaporation of the medium inside the reservoir by the generated heat (Fig. 3a3,b2,c2). After the complete evaporation of the medium, the displacements are measured 20 $\upmu$m and 68 $\upmu$m for the spheroids to which single and multiple micro-agents are attached, respectively. In order to confirm that the evaporation does not affect the attachment, a micro-agent is rotationally moved around its inserted part into the spheroid with a magnetic field of 14 mT for two minutes (Fig. 3b3). The maximum velocity for the distal end of the micro-agent is measured as 1.4 body-length/s (180 $\upmu$m/s). No detachment of the micro-agent from the spheroid is observed. Our results display that multicolor microscopy generates color-coded images in real-time for visualizing and verifying the attachment of micro-agents to the spheroids.

### Translation and rotation of HeLa cell spheroids by micro-agents attachment

**Figure 4:**
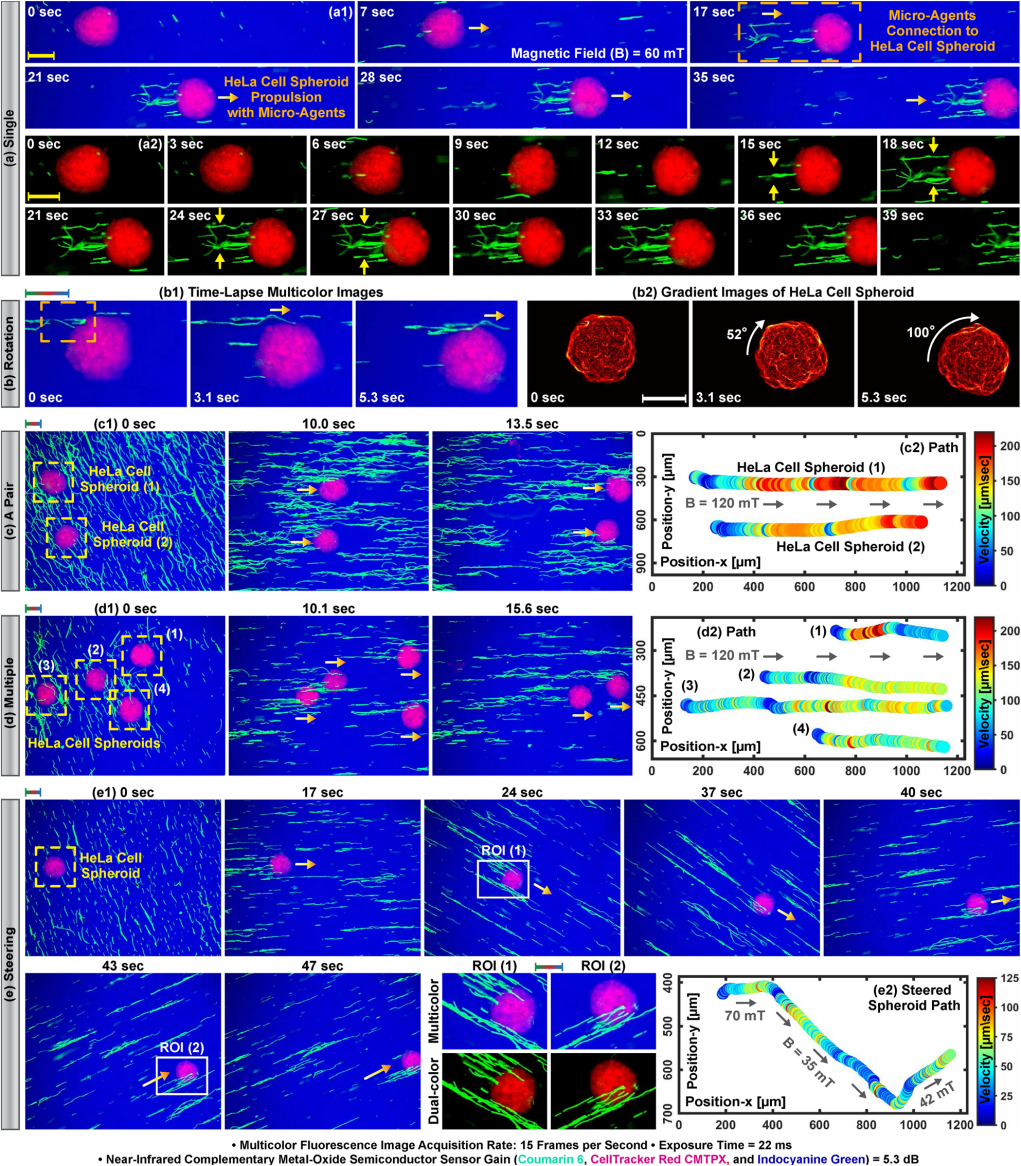
Translation of HeLa cell spheroids by magnetic micro-agents in a reservoir filled with culturing medium and indocyanine green. (**a1**) Propelling a single spheroid by micro-agents connection. (**a2**) Time-lapse dual-color microscopy for connection of micro-agents to the spheroid. (**b1**) Rotation of a spheroid caused by micro-agents propelling. (**b2**) Demonstration of the spheroid rotation by gradient images. (**c1**,**d1**) Simultaneous translation of two and multiple spheroids by micro-agents, respectively. (**e1**) Steering a spheroid propelled with micro-agents by changing the magnetic field direction. (**c2**–**e2**) Path and velocity information of the spheroids in (**c1**–**e1**), respectively. Scale bar 100 $\upmu$m. Please refer to the accompanying video (Part 1).

**Figure 5:**
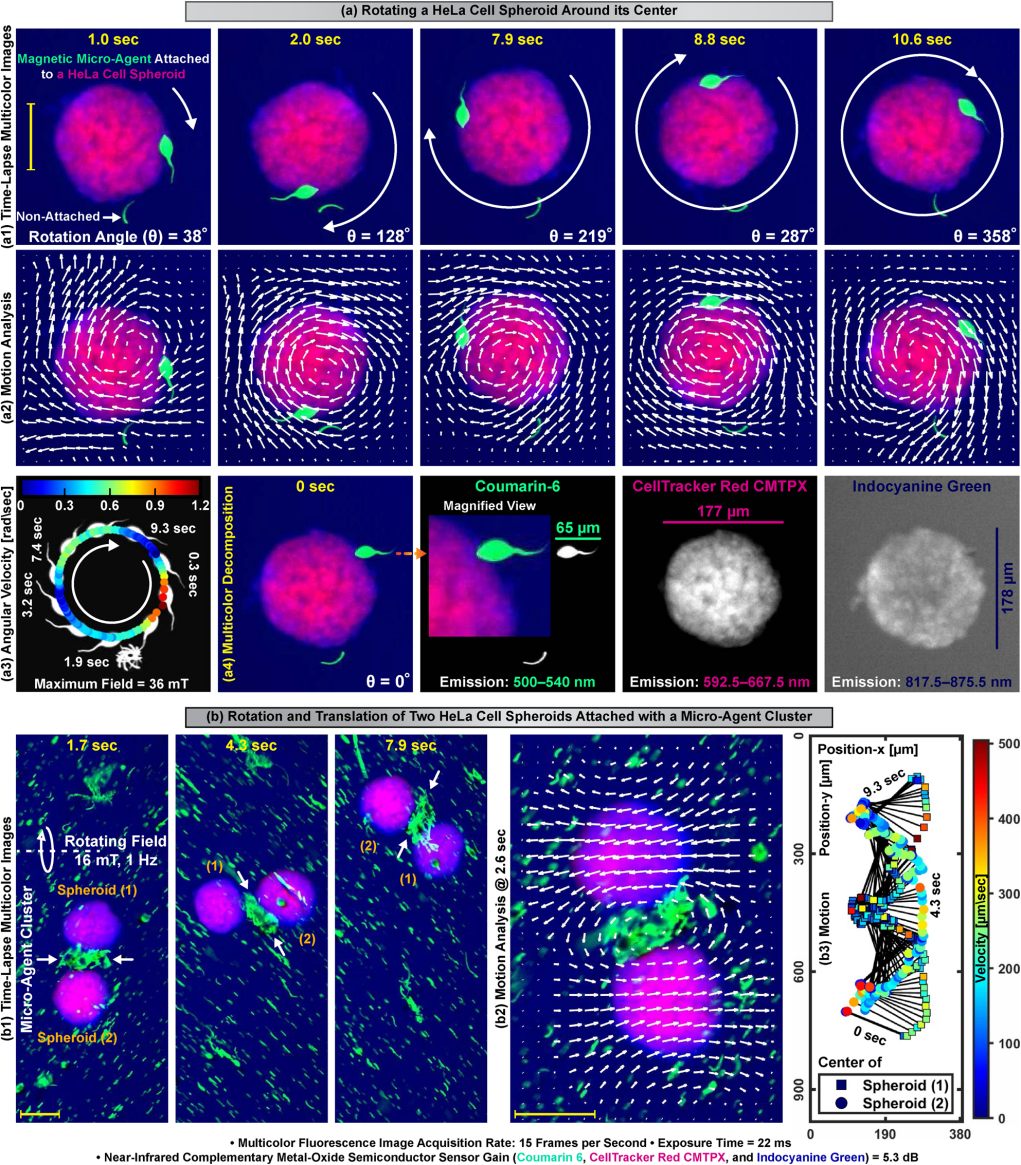
Rotation and translation of HeLa cell spheroids with micro-agents attachment in a reservoir filled with culturing medium and indocyanine green. (**a1**,**b1**) Actuation of the spheroids with single and cluster of micro-agent, respectively. (**a2**,**b2**) Vector fields overlaid on the time-lapse multicolor images show the rotational motion of the spheroids analyzed using Lucas-Kanade optical flow. (**a3**,**b3**) Motion profile and velocity information of the spheroids. (**a4**) Spectral image decomposition of the multicolor image at time zero. Scale bar 100 $\upmu$m. Please refer to the accompanying video (Part 1).

Real-time multicolor fluorescence microscopy is performed by sequential excitation of the fluorophores in a round-robin manner and synchronous individual image acquisition from 500 to 540 nm, 592.5 to 667.5 nm, and 817.5 to 875.5 nm spectrum bands. Individual images of the micro-agents labeled with coumarin 6 are acquired from the 500 to 540 nm spectrum band. The spheroids are stained with CMTPX and indocyanine green to render their visualization in 592.5–667.5 nm and 817.5–875.5 nm spectrum bands, respectively (Fig. 5a4). Thus, the simultaneous motion of the spheroids and micro-agents is able to be visualized from all the spectrum bands used in multicolor microscopy. Our micro-agents are able to mobilize HeLa cell spheroids by an external magnetic field. Figure 4a shows a spheroid propelled by attached multiple micro-agents in the reservoir containing indocyanine green in the culturing medium with a magnetic field of 60 mT. Figure 4b demonstrates the clockwise rotation of a spheroid due to imbalances in the micro-agents attachment during the propulsion. We mobilize the spheroids with micro-agents to report the performance of the real-time multicolor microscopy. The open reservoir is used as an experimental testbed since it allows translational and rotational mobility of the spheroids by micro-agents attachment. Multicolor images are formed at 15 fps, where the individual image acquisition rate is 45 fps. Figure 4c1,d1 show the forward motion of two and multiple spheroids propelled by micro-agents with a magnetic field of 120 mT, respectively. Figure 4e1 demonstrates a representative case for steering a spheroid with propulsion of the micro-agents by changing the magnetic field direction. Path information obtained by visual tracking shows the translational motions of the spheroids up to 218 $\upmu$m/s ($\approx$ 1.3 body-length/sec) in Fig. 4c2,d2,e2^41^. In order to visualize the rotational mobility, a spheroid is moved clockwise by a single micro-agent attachment with a maximum magnetic field of 36 mT (Fig. 5a1,a4). The motion information of the spheroid with micro-agent is represented as vector fields using Lucas-Kanade optical flow and shown in Fig. 5a2. The vector fields and path of the attached micro-agent display that the spheroid is rotated around its axis with a maximum angular velocity of 1.2 rad/s (Fig. 5a3). Rotational and translational mobility is visualized together by actuating two spheroids adhered with a micro-agent cluster in a 16 mT rotating magnetic field at 1 Hz (Fig. 5b1). Figure 5b2 demonstrates the motion field for the rotation of the two spheroids with the micro-agent cluster. The translational motion of the spheroids is plotted in Fig. 5b3. Our experiments show that multicolor microscopy renders visualizing the simultaneous motion of multiple spheroids and micro-agents in time-varying magnetic fields up to a translational velocity of 504 $\upmu$m/s ($\approx$ 3.4 body-length/s for a spheroid) at 15 fps. We also validate that spheroids as an organic body are able to be actuated by single, multiple, and a cluster of micro-agents in a reservoir environment. In order to immobilize a single spheroid for the attachment of micro-agents and provide a confined environment for multicolor microscopy, a microfluidic chip is developed.

### Micro-agents attachment to immobilized HeLa cell spheroids

**Figure 6:**
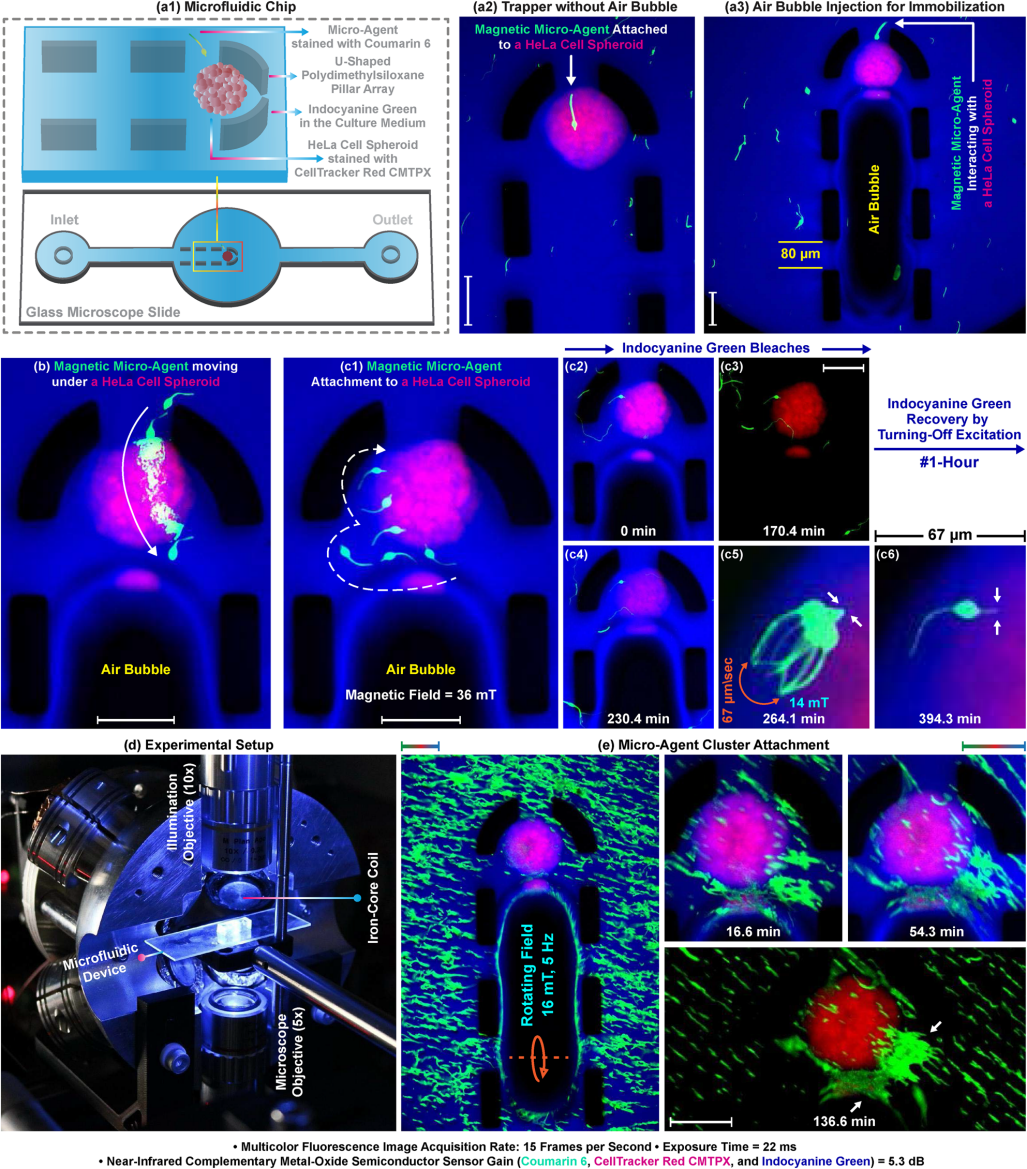
Multicolor fluorescence microscopy for visualizing the magnetic micro-agents in a microfluidic environment. (**a1**) Layout of the chip designed for immobilizing a single HeLa cell spheroid. (**a2**) Micro-agent attached to a trapped spheroid. (**a3**) Immobilizing a spheroid using an air bubble. (**b**) Overlaid image shows a micro-agent traveling under an immobilized spheroid. (**c1**) Attachment of a micro-agent to an immobilized spheroid. (**c2**–**c4**) Fluorescence recovery after photobleaching of indocyanine green. (**c5**) Attachment check by magnetically moving the micro-agent. (**c6**) Arrows indicate the part of the micro-agent inside the spheroid. (**d**) Workspace of the setup used for microscopy and magnetic actuation. (**e**) Formation of a micro-agent cluster attached to the spheroid. Scale bar 100 $\upmu$m. Please refer to the accompanying video (Part 2).

For creating a 3D simplified tumor model, a microfluidic chip that is able to immobilize a single HeLa cell spheroid with a diameter between 100 and 250 $\upmu$m is developed^42^. The chip contains a chamber constructed using a U-shaped pillar array with a spacing of 80 $\upmu$m, which blocks the escape of the spheroid and allows the entrance of micro-agents (Fig. 6a1). During the injection, the pillars trap the spheroid (Fig. 6a2). An air bubble is subsequently transferred into the chip to immobilize the spheroid by creating a pressure difference between the inside and outside of the pillar array (Fig. 6a3). Thus, the spheroid is fixed in a certain location for multicolor experiments, unlike random positioning in the open reservoir. The chip is filled with 250 $\upmu$g/ml indocyanine green in the cell culture medium to render it visible in fluorescence microscopy. The medium and micro-agents are injected without moving the spheroid owing to the pressure difference. Figure 6b demonstrates a time-lapse multicolor image for a micro-agent passing from between two pillars and under an immobilized spheroid. Figure 6c1 shows a single micro-agent attachment by puncturing an immobilized spheroid with a magnetic field of 36 mT. Dynamic imaging is performed at 15 fps for 395 min to validate that the chip maintains spheroids to which micro-agents are attached for long-term multicolor microscopy. Acquired dynamic images reveal the photobleaching resistance of indocyanine green is lower than coumarin 6 and CMTPX. Fluorescence imaging of the chip is unable due to the photobleaching of indocyanine green at minute 170 where the pixel intensity drops of the spheroid and the micro-agent are 21.2% and 1.6%, respectively (Fig. 6c2,c3). In order to recover the fluorescence imaging of the chip, the excitation light is turned off for an hour. Diffusion of non-bleached indocyanine green molecules from outside the imaging area to the inside during off time enables the multicolor microscopy again (Fig. 6c4)^43^. Fluorescence recovery is not able to be performed in the reservoir since the medium completely evaporates due to the heat produced by the excitation light (Fig. 3a3,b2,c2). The reservoir is not sealed to show the difference between open and closed microfluidic platforms in terms of multicolor image acquisition. The experiments conducted in our study demonstrate that the reservoir (as an open platform) and the microfluidic chip (as a closed platform) are able to be employed for real-time multicolor microscopy. However, evaporation of the medium inside the reservoir disables multicolor image acquisition after a certain time. An open reservoir is not a stable environment for visualizing long-term changes using dynamic multicolor imaging as long as it is not sealed with an air-tight transparent chamber to prevent evaporation. On the other hand, the chip keeps the heated medium inside it to (1) enable long-term microscopy and (2) prolong the multicolor imaging time by fluorescence recovery. In order to verify that fluorescence recovery does not affect the attachment, the micro-agent is randomly moved with a magnetic field of 14 mT between minutes 264 and 267 (Fig. 6c5). The maximum velocity for the distal end of the micro-agent is measured 67 $\upmu$m/s (1.3 body-length/s), and no detachment is observed. Figure 6c6 verifies the attachment by showing the part of the micro-agent inside the spheroid at minute 395. Multiple micro-agents are attached to an immobilized spheroid with a 16 mT rotating field at 5 Hz (Fig. 6d). We observe that the attached micro-agents form a cluster over time as a result of dipole-dipole interaction (Fig. 6e). Although a micro-agent cluster has the capability to actuate spheroids as shown in Fig 4b1, the displacement of the spheroid is measured 0 $\upmu$m for 137 min. Our experiment shows that the chip creates a static environment for multicolor microscopy by allowing multiple micro-agents to be attached without moving the spheroid. We experimentally validate that our tumor model provides a controllable and stable testbed for multicolor image acquisition of single and multiple micro-agent attachments to a spheroid. In order to verify that multicolor microscopy can visualize micro-agents in vascularized environments, a vascular network on a microfluidic system is used as an experimental testbed.

### Micro-agents inside a perfusable vascular network

**Figure 7:**
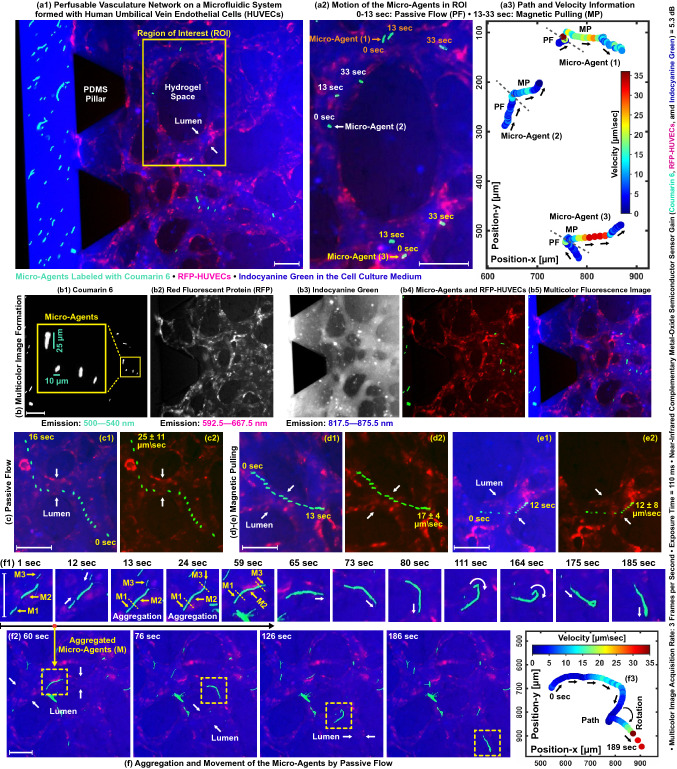
Multicolor fluorescence microscopy for visualizing the magnetic micro-agents in a perfusable vasculature network. (**a1**) Multicolor image of the micro-agents labeled with coumarin 6 in a vasculature formed with RFP-HUVECs on a microfluidic system filled with culture medium and indocyanine green. (**a2**) Movement of the micro-agents by passive flow and magnetic pulling with a field of 70 mT. (**a3**) Path and velocity of the micro-agents. (**b**) Multicolor image formation by overlapping the acquired spectrally-different images. (**c1**,**d1**–**e1**) Movement of the micro-agents in a lumen by passive flow and magnetic pulling with a field of 42 mT, respectively. (**c2**–**e2**) Merged fluorescence images of the micro-agents and lumens. (**f1**,**f2**) The aggregation and movement of the micro-agents in a vasculature by passive flow, respectively. (**f3**) Velocity and path of the aggregated micro-agents. Scale bar 100 $\upmu$m. Please refer to the accompanying video (Part 3).

In vitro perfusable vascular network is engineered by co-culture of RFP-HUVECs and bone marrow-derived mesenchymal stem cells in a microfluidic system. For multicolor microscopy, 250 $\upmu$g/ml indocyanine green in the cell culture medium and micro-agents are injected into the system. Figure 7a1 depicts a static multicolor image of the microfluidic system featuring the engineered vascular network and micro-agents. Figure 7a2 demonstrates the magnified view of the ROI defined on the multicolor image and highlights the movement of micro-agents in the vascular network by passive flow and magnetic pulling with a field of 70 mT. Micro-Agent (1) freely moves along the magnetic field, whereas Micro-Agents (2) and (3) hit the lumen walls after 33 s. Our experiment display that micro-agents perfuse into the vascular network via lumen openings and can be simultaneously tracked (Fig. 7a3). Figure 7b shows representative multicolor image formation and verifies that micro-agents, vascular network, and microfluidic system are spectrally resolved in three distinct spectrum bands. The structural morphology of the vascular network is visualized from dual spectrum bands (592.5–667.5 nm and 817.5–875.5 nm) owing to the diffusion of indocyanine green in the culturing medium (Fig. 7b2,b3). This enables complete visualization of the vasculature structure and clearly delineates the hydrogel space (Fig. 7b4,b5). The mobility of the micro-agents inside the vascular network is visualized in three sets of multicolor experiments under non-cell culture conditions. In all experiments, multicolor images are acquired at 3 fps to minimize the photodamage on RFP. The higher image acquisition rate is achievable with incubation conditions since RFP-HUVECs have an active good working metabolism to rebuild the fluorescence signals. The first experiment is conducted to visualize a micro-agent moved by the passive flow (Fig. 7c). The average velocity of the micro-agent is computed as 3.3 body-length/sec (25 $\upmu$m/s). The second experiment is designed to visualize micro-agents magnetically pulled against the passive flow with a field of 42 mT. Figure 7d,e shows the overlaid time-lapse images of the micro-agents movement with the average velocity of 1.4 body-length/s (17 $\upmu$m/s) and 2.5 body-length/s (12 $\upmu$m/s), respectively. In the third experiment, the aggregation of three micro-agents utilizing electrostatic forces is visualized (Fig. 7f1). The aggregated micro-agents are moved by the passive flow with the average velocity of 0.04 body-length/s (4 $\upmu$m/s) (Fig. 7f2,f3). Acquired multicolor images reveal that no disaggregation occurs for 186 s (Fig. 7f1,f2). Our imaging experiments demonstrate that multicolor microscopy: (1) provides spatiotemporal resolution for quantitative analysis of the micro-agents inside the vasculature, (2) allows image acquisition of the micro-agents with a minimum length of 5 $\upmu$m, (3) enables unambiguous color-coding of both micro-agents and engineered vascular morphology in real-time. Next, we report the multicolor microscopy performance by visualizing micro-agents inside and outside the natural blood vessels.

### Micro-agents inside chorioallantoic membrane vasculature

**Figure 8:**
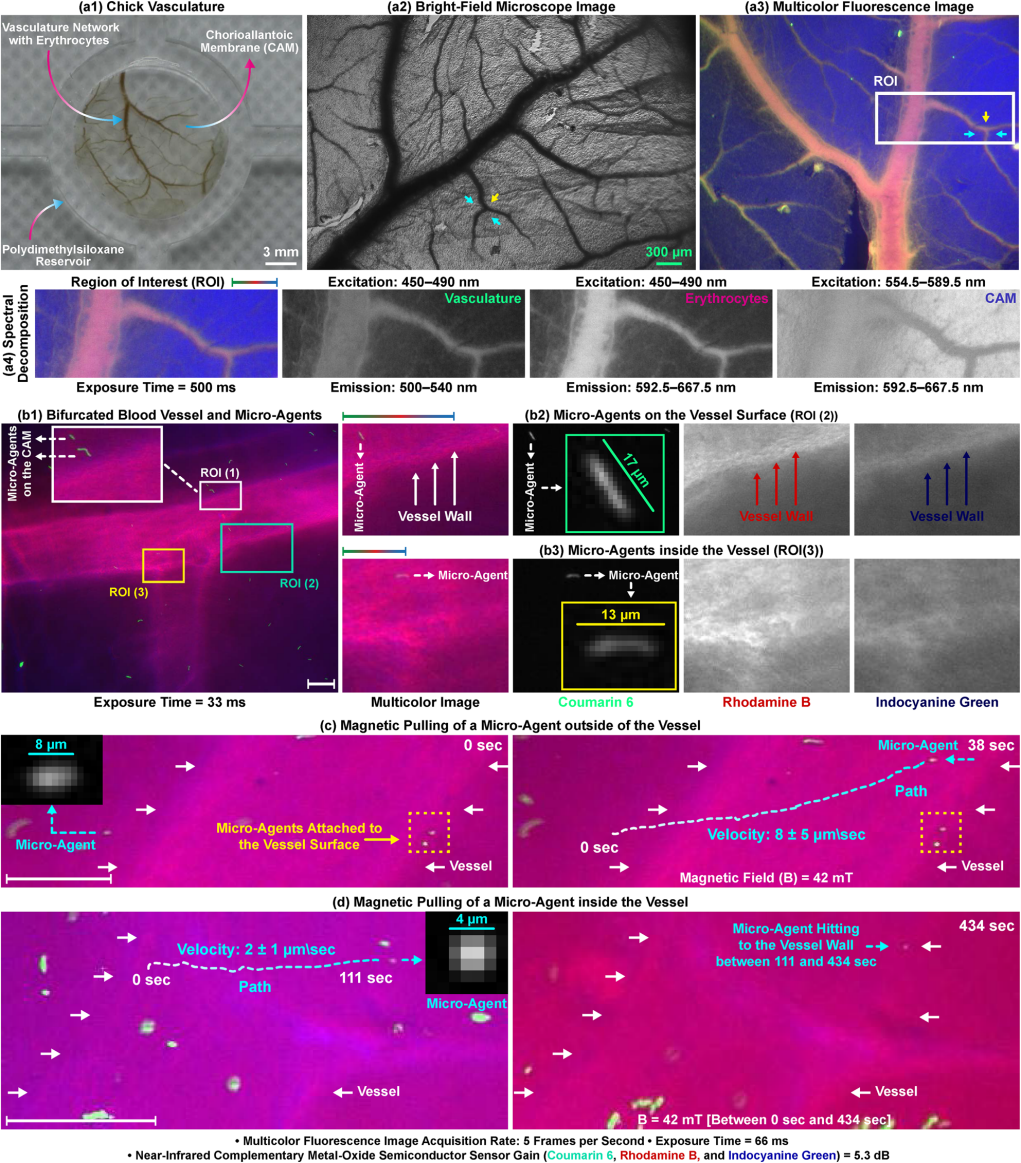
Multicolor fluorescence microscopy for visualizing the magnetic micro-agents in ex ovo chicken chorioallantoic membrane. (**a1**) Prepared sample for vasculature network visualization utilizing autofluorescence property of erythrocytes. (**a2**,**a3**) Bright-field and multicolor images of the network, respectively. (**a4**) Spectral decomposition of ROI on (**a3**). (**b1**) Multicolor image of the micro-agents labeled with coumarin 6 and a vessel containing Rhodamine B and indocyanine green. ROI (1)–(3) on (**b1**) showing the micro-agents on the membrane, on the vessel surface, and inside the vessel, respectively. (**b2**,**b3**) Magnified view and spectral decomposition of ROI (2) and (3), respectively. (**c**,**d**) Magnetic movement of the micro-agents outside and inside the vessels, respectively. Scale bar 100 $\upmu$m. Please refer to the accompanying video (Part 4).

Ex ovo chorioallantoic membrane (CAM) vasculature containing the hierarchical multiscale blood vessels is used to visualize the micro-agents in a native environment (Fig. 8a1). Figure 8a2,a3 show the structural morphology of the vasculature visualized using bright-field as a reference and multicolor microscopy with autofluorescence of erythrocytes, respectively. The multicolor image is formed by spectral unmixing of three main cellular features: vasculature, erythrocytes, and CAM (Fig. 8a4). We perfuse the CAM using DPBS to completely remove the free-floating erythrocytes within the vasculature structures. This decellularized vascular structure allows free movement of micro-agents as well as multiple fluorophore solutions. For staining, vasculature is placed in a Petri dish containing 250 $\upmu$g/ml indocyanine green in culture medium, 100 $\upmu$g/ml Rhodamine B in water, and the micro-agents for an overnight period. Before the imaging experiments, the dish containing the stained sample is washed with DPBS by manual shaking for 15 min to prevent the aggregation of the micro-agents on the CAM. A circular part with a diameter of 15 mm is cut out from the sample and transferred onto a PDMS reservoir for multicolor microscopy. The representative static multicolor image of a decellularized bifurcated blood vessel and the micro-agents after DPBS washing is shown in Fig. 8b1. ROI (1)–(3) defined on the multicolor image plane highlight micro-agents on the chorioallantoic membrane, on the vessel wall, and inside the vessel, respectively. In order to reveal details about micro-agents and vessels, the magnified view of ROI (1)–(3) are shown in Fig. 8b1–b3, respectively. Spectral image decomposition of ROI (2) and (3) verifies that the micro-agents and the vessel are imaged from different spectrum bands, and there is a contrast difference between CAM and vessel wall (Fig. 8b2,b3). The contrast difference decreases by 90% since heterogeneous pores present on the vessel wall allow diffusion of Rhodamine B and indocyanine green (Fig. 8c,d). Real-time multicolor images are acquired at 5 fps with 66 ms exposure time, where the micro-agents are pulled with a magnetic field of 42 mT. The average velocity of the micro-agents moving outside and inside the vessel is computed as 1 body-length/s length/s (8 $\upmu$m/s) and 0.5 body-length/s length/s (2 $\upmu$m/s), respectively (Fig. 8c,d). The micro-agent inside the vessel is pulled until it reaches the wall. The displacement of the micro-agent after it reaches the vessel wall is measured as 0 $\upmu$m for 5 min, whereas the micro-agents outside travel along the magnetic field (Fig. 8d). Our experiment demonstrates that multicolor microscopy provides real-time visualization of the micro-agents inside the natural blood vessels.

## Conclusions

In this work, we demonstrate multicolor fluorescence microscopy to visualize polymeric micro-agents within 3D tumor-on-a-chip and vasculature models for the envisioned application of targeted drug delivery. Compared to the imaging techniques used in the literature, micro-agents and surroundings are spectrally resolved in three distinct spectrum bands, and color-coded visualization of the models is acquired. We experimentally validate that multicolor microscopy allows spectrally-different image acquisition of micro-agents at varying aspect ratios, organic bodies, surrounding media up to 15 fps, and sequential motion detection up to 504 $\upmu$m/s from each spectrum band. As a result of color-coding, an increased understanding of the identification and discrimination of each micro-component is obtained. Our measurements show that multicolor microscopy delineates the spatial compartments in real-time for analyzing and verifying the functionality of the micro-agents. We expect real-time multicolor microscopy to accelerate the translation of micro-agents into practice by enabling the required clear visualization.

## Methods

### Micro-agent fabrication using electrospinning

Electrospinning setup for fabrication of micro-agents is constructed using a high-voltage power supply (60C24-P250-I5, Advanced Energy, USA) and a syringe pump (NE-4000, New Era Pump Systems, USA) (Fig. 1a). A data acquisition unit (34972A, 34901A, 34907A, Keysight, USA) is used to both transfer control signals to the high-voltage power supply and collect feedback data. The setup is placed inside an air-tight enclosure to ensure reproducible fabrication by providing the same air convection condition. Polymer solution for the electrospinning consists of polystyrene pellets (430102-1KG, Sigma-Aldrich, USA) as carrier polymer, anhydrous N,N-Dimethylformamide (DMF) as solvent (227056-1L, Sigma-Aldrich, USA), coumarin 6 (442631-1G, Sigma-Aldrich, USA) as a hydrophobic fluorophore, and iron oxide nanoparticles ($\hbox {Fe}_{3}\hbox {O}_4$) (637106-25G, Sigma-Aldrich, USA) as magnetic material^44,45^. For fabrication of continuous beaded and straight fibers, solutions with 30.02% (19.8% polystyrene, 10.0% $\hbox {Fe}_{3}\hbox {O}_4$, 0.2% coumarin 6) and 45.25% (30.1% polystyrene, 15.0% $\hbox {Fe}_{3}\hbox {O}_4$, 0.2% coumarin 6) weight-to-volume ratio in DMF are prepared, respectively. $\hbox {Fe}_{3}\hbox {O}_4$ and DMF are first sonicated for 30 min to disaggregate the particles. Polystyrene pellets and coumarin 6 are subsequently added to the mixture and blended using a roller mixer (LLG-uniRoller 6, LLG-Labware, Germany) for 12 h to achieve a homogeneous solution. For the electrospinning process, the polymer solution is placed in a plastic syringe and connected through a Teflon tubing to an 18-gauge blunt-tip needle (TS18SS-15, Adhesive Dispensing Ltd., UK). Accelerating voltage, solution feed rate, and tip-to-collector distance are set as 14 kV, 2.5 mL/h, and 16 cm, respectively. 0.04 mL polymer solution is electrospun at once, and fiber meshes are deposited on a grounded aluminum foil at 24 $^{\circ }$C and 22% humidity. The solvent residue on the electrospun fibers is removed by drying at room temperature for 12 hours. Figure 1b1,b2 show the morphologies of the fiber meshes imaged with scanning electron microscopy (JSM-7200F, JEOL, Japan). Deposited fiber meshes are cut into 2-3 mm pieces using a scalpel and then immersed into a glass vial containing 1% (volume/volume) Tween 80 (P4780-100ML, Sigma-Aldrich, USA) in Milli-Q water. Fluorescent and magnetic micro-agents are obtained by cutting the fibers using a sonicator (2510, Branson, USA) for 2 h (Fig. 1c1). The polymer grinding process using sonication randomly shortens the continuous fibers to obtain micro-agents. The electrospinning setup is also designed for depositing the fibers on the collector with random orientation. This fabrication technique is selected to enable the validation of multicolor image acquisition using micro-agents with random size distribution. In order to obtain micro-agents with desired sizes, synthesized fibers can be aligned with a drum collector and cut using a laser^46^. Additionally, the electrospraying process ensures the fabrication of bead-shaped micro-agents with narrow size distribution. The setup is able to be used for electrospraying without any modification^47^. Figure 1c2 shows fluorescence images of the fabricated micro-agents. The surface profile of a micro-agent characterized using confocal microscopy (S Neox, Sensofar, Spain) is shown in Fig. 1c3. In order to determine optimal excitation and emission wavelength ranges for fluorescence microscopy, the spectrum of the micro-agents is characterized using a spectrofluorometer (FP-8300, Jasco, Japan).

### Spectrum analysis

The solution for the spectrum analysis is prepared by dissolving 2.11 mg coumarin 6 in 5 ml DMF. 10 $\upmu$l solution is diluted with 690 $\upmu$l DMF in a quartz cuvette (CV10Q700F, Thorlabs, USA) for the measurement. Ultraviolet-visible excitation and emission spectra, the wavelength range between 200 and 900 nm, is measured using 2D spectrum analysis (Fig. 1d1). Peak excitation and emission wavelengths for coumarin 6 in DMF are measured as 461 nm and 505 nm, respectively. 3D spectrum analysis is performed to measure emission spectrum at varying excitation wavelengths. The solution is excited between 350 nm and 500 nm with a 1 nm increment and emitted fluorescent photons are collected in the wavelength range of 450 nm and 600 nm (Fig. 1d2,d3). Our measurement shows that fluorescence photons with maximum intensity are collected when the excitation and emission are performed with 436–477 nm and 497–518 nm spectrum bands, respectively. According to the spectrum analysis, 450–490 nm and 500–540 nm bands are selected to excite coumarin 6 and collect emitted fluorescent photons for visualization of the fabricated micro-agents, respectively. Figure 1e1 shows an example of single-band fluorescence microscopy for magnetic movement of a micro-agent. Multicolor microscopy is performed to visualize the micro-agents in a tumor environment containing HeLa cell spheroids with a diameter of 200 $\upmu$m.

### HeLa cell spheroids formation

HeLa cells are cultured in Dulbecco’s modified Eagle’s medium (11-965-092, Fisher Scientific Ltd. Canada), supplemented with 10%, fetal bovine serum (F7524-500ML, Sigma-Aldrich, USA), and 1% (volume/volume) penicillin-streptomycin (15-140-122, Thermo Fisher Scientific Inc., USA)^29^. Cells in 2D are cultured and passaged at 80% confluence and medium change is performed every 48 hours. For the formation of 3D cancer spheroids, cells are trypsinized, resuspended to a density of $2\times {10}^6$ cell/ml, and seeded in 3% (weight/volume) agarose (16500500, ultra-pure agarose, Invitrogen, USA) microwell arrays resulting in average 267 cells per spheroid. Half medium change is done daily. At day 3, spheroids are collected and stained with $10\,\upmu \hbox {M}$ CellTracker Red CMTPX (C34552, Thermo Fisher Scientific Inc., USA) by 45 min incubation in a serum-free medium and washed twice with cell culture medium. Both 2D and 3D cultures are done in a humidified atmosphere with 5% carbon dioxide at 37$^\circ$C. For long-term storage, 3D spheroids stained with CMTPX are fixated at room temperature with 4% formaldehyde (F8775-25ML, Sigma-Aldrich, USA) for 15 min and then washed twice with Dulbecco’s phosphate-buffered saline (DPBS). For imaging experiments, the spheroids are immobilized and maintained using the developed microfluidic chip (Fig. 6a).

### Microfluidic chip fabrication

The standard soft-lithography method is used to fabricate negative mold of the chips with the height of 187 $\upmu$m using SU-8 photoresist^48^. The salinization process is performed for easy removal of the polydimethylsiloxane (PDMS) layer by improving the surface hydrophilicity of the mold. Degassed 10:1 PDMS and curing agent (Sylgard 184 Silicon Elastomer Kit, Dow Corning, USA) mixture is poured over the mold and cured at 70 $^\circ$C in the oven for 12 h. After the cured PDMS layer is removed, inlet and outlet ports are punched. The chip fabrication is completed by bonding the PDMS layer on a microscope glass using plasma oxidation. The bonding strength is improved by baking the chip on a hot plate at 70 $^\circ$C for 4 h. A single cell spheroid is first transferred into the chip and immobilized by an air bubble injection. Micro-agents are mixed with 250 $\upmu$g/ml indocyanine green (I2633-25MG, Sigma-Aldrich, USA) in the culture medium and subsequently injected into the chip by pipetting. Thus, a 3D tumor model is created for multicolor image acquisition (Fig. 6a3). In order to show that multicolor microscopy provides spectrally resolved images for the micro-agents in vascularized models, a perfusable vascular network is formed on a microfluidic channel.

### 3D co-culture in a microfluidic system for in vitro vascularization

Red fluorescence protein-human umbilical vein endothelial cells (RFP-HUVECs) (cAP-0001, Angio-Proteomie, USA) are cultured in 2D with endothelial cell growth medium-2 (EGM-2 medium) (Endothelial Cell Growth Medium 2 Kit, C-22111, Promo Cell, USA) with 1% (volume/volume) (v/v) penicillin-streptomycin. In parallel, mesenchymal stem cells (PT-2501, Lonza, USA) are cultivated in 2D in minimum essential medium eagle-alpha modification (22571020, Thermo Fisher Scientific, USA) supplemented with 10% (v/v) fetal bovine serum (F7524v, Sigma-Aldrich, USA), 2 mM l-glutamine (35050061, Thermo Fisher Scientific, USA), 0.2 mM ascorbic acid (A8960-5G, Sigma Aldrich, USA), 1% (v/v) penicillin-streptomycin (15-140-122, Thermo Fisher Scientific, USA) and 1 ng/ml fibroblast growth factor-basic (PHG0261, Thermo Fisher Scientific, USA). Both cell types are cultured in a humidified atmosphere with 5% carbon dioxide at 37 $^{\circ }$C and are used between passages 4 and 6. The cells are cultured till 80% confluency prior to detachment and mixed in a concentration of $10\times 10^{6}$ cells/ml (1 to 1 ratio) in a fibrin hydrogel in a final concentration of 3 mg/ml (Fibrinogen, 0215112201, MP Biomedicals, USA) and 3 U of thrombin (0215416301, MP Biomedicals, USA). The cell-matrix mixture is injected into the hydrogel chamber of the gel loading port of the microfluidic system and is allowed to polymerize in the incubator at 37 $^{\circ }$C for 15 min. Before, the microfluidic surface area is functionalized to promote strong hydrogel interaction with the PDMS and glass by incubating the microfluidic chip after plasma oxidation bonding for 4 h with poly-d-lysine hydrobromide (P1024-10MG, Sigma-Aldrich, USA) with following washing steps with sterile Milli-Q water. After hydrogel polymerization, the gel loading ports are blocked with stripes of standard PCR plate sealing foil to avoid fluidic leakages and the flow channels next to the hydrogel channel are filled with EGM-2 medium. The pressure drop of 5 Pa to create the mechanical stimuli for the in vitro vascularization is established by connecting two syringe pumps (AL-1600, New Era Pump Systems, USA) on vertically opposite sides. An overview of the microfluidic system for in vitro vascularization creation is illustrated in Supplementary Fig. 1. The inlet port is used to inject the EGM-2 medium and the outlet port to pull it out while the fluidic ports are blocked with PDMS after the initial filling. By applying pre-determined flow rates with EGM-2 medium by flow studies performed using COMSOL Multiphysics (version 5.5, COMSOL AB, Sweden). The pressure drop is maintained for 7 days before the experiments. For multicolor microscopy, 250 $\upmu$g/ml indocyanine green in the cell culture medium and micro-agents are injected in the microfluidic system. Figure 7a1 shows the micro-agents in the engineered vascular network. In order to visualize micro-agents in natural vessels, ex ovo chicken chorioallantoic membrane is used as a testbed.

### Chicken vasculature

White Leghorn chick embryos are incubated at 38 $^\circ$C and 65% humidity throughout the culturing process. At day 10, the egg contents are cracked into 60 mm cell culture Petri dishes^49^. Egg albumin and yolk contents are removed using viscous liquid handling pipettes. The vasculature is washed three times using ice-cold DPBS (14190250, Thermo Fisher Scientific, USA) solution and then fixated in 4% paraformaldehyde (1004965000, Sigma-Aldrich, USA) overnight at 4 $^\circ$C for long-term storage. This is followed by incubation with 0.3% Triton X-100 (T8787-250ML, Sigma-Aldrich, USA) in DPBS for 30 minutes and washing three times using ice-cold PBS solution. For multicolor microscopy, the vasculature is first decellularized (erythrocytes removed) and subsequently stained with Rhodamine B (K94900-25, Labshop, the Netherlands) and indocyanine green.

### Multicolor fluorescence microscopy and magnetic movement

Real-time widefield multicolor microscopy is performed by excitation of the fluorophores from three-different spectrum bands in a round-robin manner^29^. The emitted fluorescence light is synchronously collected to acquire 8-bit grayscale individual images with 1280 $\times$ 1024 pixels. 10x and 5x long working distance objectives (Plan Apo, Mitutoyo, Japan) are used to focus the excitation light on the samples and collect the emitted fluorescent photons, respectively. Coumarin 6 and indocyanine green are excited from 450 to 490 nm and 750 to 800 nm spectrum bands, respectively. The emitted light is collected from 500 to 540 nm for coumarin 6 and 817.5 to 875.5 nm for indocyanine green, respectively. CMTPX, RFP-HUVECs, and Rhodamine B are excited from the 554.5 to 589.5 nm band, and the emitted light is collected in the wavelength range of 592.5 to 667.5 nm. The intensity values in the individual images acquired from 500 to 540 nm, 592.5 to 667.5 nm, and 817.5 to 875.5 nm spectrum bands are represented using black-green, black-red, black-blue color maps, respectively (Figs. 5a4, 7b, 8a4). Multicolor images are formed by overlapping the color-converted individual images acquired in one round. An orthogonal array of four iron-core electromagnetic coils with a cut-off frequency of 110 Hz is placed in the microscope’s working space to move the micro-agents using the rotational field (Fig. 6d). For heat dissipation, the coil array is mounted on an aluminum holder using tapered collars. The coils are covered with copper tape for heat transfer enhancement and electrical isolation. Switchable amplifiers supplied with a dc power supply (EA-PS 5040-20A, EA Elektro-Automatik, Germany) are used to power the coils^50^. A signal generator (4050B, BK Precision, USA) is used to generate reference signals for the rotating field frequency. The signals are distributed to a corresponding coil driver using a demultiplexer interface (74HC4052, Texas Instruments, USA) by falling edge detection using a microcontroller. The maximum magnetic field produced by the coil array is measured as 30 mT using a teslameter (3MH3A, Senis AG, Switzerland). The magnetic pulling force is generated using two neodymium-iron-boron permanent magnets (S-45-30-N, Supermagnete, Germany). The maximum magnetic field and gradient generated by the magnets are measured as 120 mT and 5 T/m, respectively.

### Ethical approval

According to the Dutch animal care guidelines, IACUC approval for chicken embryo experimentation is not necessary unless hatching is expected. Moreover, only experiments with chick embryos of development EDD14 and older need IACUC approval. The embryos used in this study were all in the early stages of embryo development (EDD10). Fertilized chicken eggs used in this study were purchased from approved poultry egg farms in the Netherlands. RFP-HUVECs culture studies were approved by Dutch Ministry and experiments were performed under ML-1 laboratory environment, followed by the university policies and standard protocol.

## Supplementary Information

Supplementary Information 1.

Supplementary Information 2.

Supplementary Information 3.

## Data Availability

All data generated or analyzed during this study are included in this published article and its supplementary information files.
