# Supplementary material for: Visualization of micro-agents and surroundings by real-time multicolor fluorescence microscopy
Source: Sci Rep. 2022 Aug 4;12:13375. doi: 10.1038/s41598-022-17297-7 (PMC9352757; doi:10.1038/s41598-022-17297-7)
Supplement: Supplementary file 2 — Supplementary Information 2. [file 41598_2022_17297_MOESM2_ESM.pdf]

Visualization of Micro-Agents and Surroundings by Real-Time Multicolor Fluorescence Microscopy Supplementary Material

Mert Kaya[1,2,*], Fabian Stein[3], Prasanna Padmanaban[3], Zhengya Zhang[2], Jeroen Rouwkema[3], Islam S. M. Khalil[1], and Sarthak Misra[1,2]

[1]Surgical Robotics Laboratory, Department of Biomechanical Engineering, University of Twente, 7522 NB Enschede, The Netherlands
[2]Surgical Robotics Laboratory, Department of Biomedical Engineering and University Medical Centre Groningen, University of Groningen, 9713 AV Groningen, The Netherlands
[3]Vascularization Laboratory, Department of Biomechanical Engineering, University of Twente, 7522 NB Enschede, The Netherlands
[*]m.kaya@utwente.nl

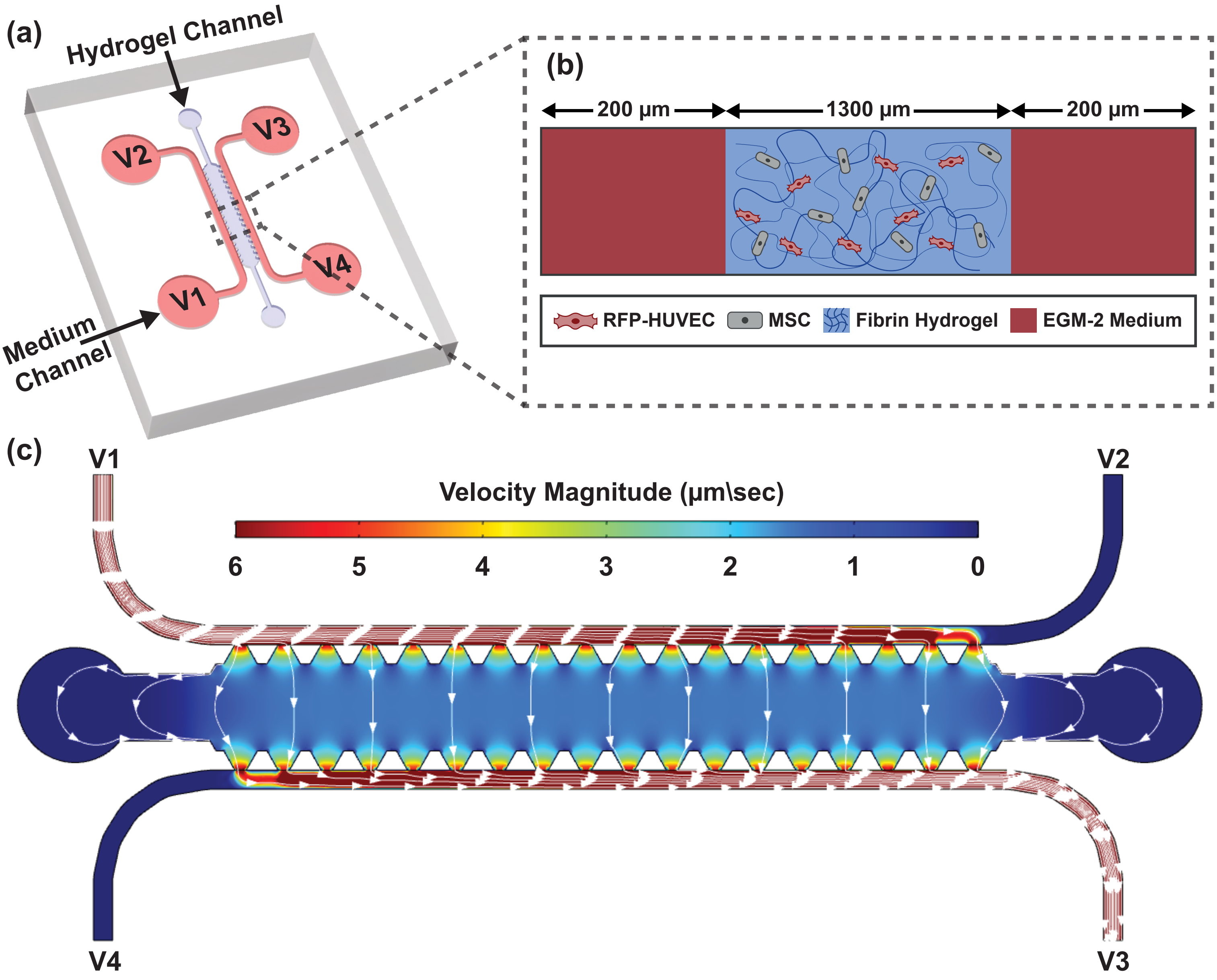

Supplementary Figure 1. General overview of the microfluidic system to create an *in vitro* vascularization for the mobility of micro-agents. (a) Schematic representation of the microfluidic system to illustrate the hydrogel filling ports and the different flow channel access points. The hydrogel channel is filled with a fibrin hydrogel with co-cultured red fluorescence protein - human umbilical vein endothelial cells (RFP-HUVECs) and mesenchymal stem cells (MSC) (b) and perfused with endothelial cell growth medium-2 (EGM-2 medium) for 7 days under constant interstitial flow. (c) Velocity heat map of the flow simulation using COMSOL Multiphysics (version 5.5, COMSOL AB, Sweden) for displaying the uniform interstitial flow across the fibrin hydrogel for stimulating the vascular formation of the embedded co-culture cells.
